# Youth with chronic health problems: how do they fare in main-stream mentoring programs?

**DOI:** 10.1186/s12889-017-5003-3

**Published:** 2018-01-05

**Authors:** Ellen L. Lipman, David DeWit, David L. DuBois, Simon Larose, Gizem Erdem

**Affiliations:** 1 Department of Psychiatry and Behavioural Neurosciences, Offord Centre for Child Studies, McMaster University, McMaster Innovation Park, Suite 201A, 1280 Main St. West, Hamilton, ON L8S 4K1 Canada; 2 Department of Epidemiology and Biostatistics, Western University, 1151 Richmond St, London, ON N6A 3K7 Canada; 3 Institute for Health Research and Policy, University of Illinois, 1200 W Harrison St, Chicago, IL 60607 USA; 4 Faculty of Education, 2325 Rue de l’Université, Ville de Québec, QC G1V 0A6 Canada; 5 Department of Psychology, Koc University, Rumelifeneri Mahallesi, Rumelifeneri Yolu, 34450 Sarıyer/İstanbul, Turkey

## Abstract

**Background:**

Youth with chronic physical health problems often experience social and emotional problems. We investigate the relationship between participation in the Big Brothers Big Sisters of Canada community-based mentoring program**s** (BBBS) and youth social and mood outcomes by youth health status.

**Methods:**

Youth newly enrolled in BBBS were classified by health status (one or more chronic physical health problems without activity limitation, *n* = 191; one or more chronic physical health problems with activity limitation, *n* = 94; no chronic health problem or activity limitation, *n* = 536) and mentoring status (yes/no) at 18 month follow-up. Youth outcomes measured at follow-up were social anxiety, depressed mood, and peer self-esteem.

**Results:**

Youth with chronic health problems and activity limitation were more likely to live with two biological parents, use mental health or social services, and have parents who reported difficulties with depressed mood, social anxiety, family functioning and neighbourhood problems. At 18 month follow-up, mentored youth in this health status group experienced fewer symptoms of social anxiety and higher peer self-esteem compared to non-mentored youth. Mentored youth with chronic health problems without activity limitation and mentored youth with no health problems or limitations did not show significant improvements in social anxiety and peer self-esteem. Regardless of their health status, mentored youth reported fewer symptoms of depressed mood than non-mentored youth.

**Conclusions:**

Youth with chronic health problems, particularly those with activity limitation as well, demonstrate a capacity to experience social and mood benefits associated with mentoring.

## Background

Medical advances in pediatrics have enabled children and adolescents (henceforth youth) with chronic health problems to survive longer. Prevalence estimates have doubled [1], with up to one in four youth experiencing a chronic illness [2]. Some of these youth also experience difficulties with school attendance and performance (e.g., [3, 4]), as well as social, emotional and behavioral problems [5–11]. Meta-analyses examining risk of these problems among youth with chronic health problems show significantly elevated internalizing (*g* = .47), externalizing (*g* = .22) and total behavior problems (*g* = .42) compared with healthy peers [5]. As youth with chronic health problems may experience a spectrum of physical health, mental health and social difficulties, strategies to assist them should be wide-ranging and have the potential to assist broadly.

We know little about how best to assist with emotional, behavioral and social difficulties among youth with chronic health problems. A systematic review identified fourteen diverse programs to assist with positive youth development (competence, confidence, character, social connection, compassion) for youth with childhood-onset chronic diseases [12]. Programs offered some or all of leadership activities, life skills development and sustained relationships with adult mentors and evaluated medical, health care transition, and other varied psychosocial outcomes. While some programs resulted in better quality of life and self-esteem, others did not. Methodologic rigour of the included studies varied, with concerns about lack of a control group, small sample sizes and limited follow-up noted. Samson-Daly and colleagues reviewed psychological interventions for individuals with chronic illness [13]. However the focus was on a broader age span (10-30 years old), only four studies examined emotional/peer support and the only variable measured was peer support. Examination of six mentoring and peer-led programs using social support to improve quality of life of adolescents with chronic illness [14] identified social support (e.g., mentoring by peers/adults) as important [14]. Studies of the psychological and social impact of specialized camps for children with chronic illness [15, 16] concluded these may offer some short-term psychological benefits but little evidence of sustained impact and identified methodologic concerns.

Another approach to assisting youth with chronic health problems manage social and emotional/behavioral problems is involvement in mainstream programs that can assist all youth with these issues. Meta analyses of youth mentoring programs [17, 18] have shown that youth paired to an adult mentor experience significant improvements in psychological, behavioral and social outcomes compared to non-mentored youth. No study has examined the impact of participation in a mainstream mentoring program for youth with chronic health problems.

We investigate the relationship between mentoring status and selected mental health outcomes for youth in three health status groups (one or more chronic physical health problems and no activity limitation; one or more chronic physical health problems and activity limitation; no chronic physical health problem or activity limitation) participating in a national study of Canadian Big Brothers Big Sisters (BBBS) community-based mentoring program.

## Methods

### Program description

The BBBS community-based mentoring programs [19] provide youth with a one-to-one relationship with an adult volunteer mentor. For one year (minimum), mentors spend 2-4 h (average) each week with their mentee in recreational, skill, or career-oriented activities. Mentors attend a training session prior to being matched. To determine a match, caseworkers interview qualified families and mentors to assess common interests, preferences and mentor ability to meet youth needs. Caseworkers contact families and mentors at least monthly for six months then bi-monthly until 12 months then quarterly. Caseworkers may provide advice on match-related problems or information on organizational events. Enrolled youth are assigned to a waiting list until they are paired to a mentor.

Sample Selection and Recruitment:

Twenty agencies were recruited by the national BBBS office to participate. Most (80%) were in metropolitan centers and chosen based on a long history of operation, large annual caseloads, and culturally diverse clients. To qualify for the study, families were required to be new admissions, have passed the agency’s qualifying assessment, and youth had to be 6-17 years old. One youth was randomly selected to participate in families with more than one eligible youth. Parents/guardians had to have primary parenting responsibility for the youth. Across all agencies, 1279 families meeting the study eligibility criteria were approached by BBBS caseworkers, and 997 (78%) agreed to participate and completed a baseline assessment. Detailed study methodology is reported elsewhere [18]. The study was approved by the Centre for Addiction and Mental Health Research Ethics Board.

The initial sample for the current study consisted of 859 youth with mentoring status information at the 18 month follow-up. Three-quarters (75%) had a previous or ongoing mentoring relationship; 46% for at least 12 months [20], the minimum period of BBBS mentor commitment expected. Information on the mentoring status of youth not completing an 18 month follow-up (*n* = 212/859) (scheduling difficulties 71%, drop out 29%) was obtained from earlier or later follow-ups for the missing 18 month values. Youth not completing 18 month follow-up were compared to completers on baseline demographics, personal characteristics, and environmental factors. Results revealed non-completers were older (*OR* = 1.10, *p* < .01), had more recent family moves (*OR* = 1.08, *p* < .05), and younger parents (*OR* = 1.03, *p* < .05).

To assess youth chronic health problems, parents were asked if their child currently had any long-term illness or medical condition. Parents replying yes were asked to list the specific conditions. Dominant conditions included breathing difficulties/asthma (55%), allergies (29%), speech, hearing/vision problems (9%) and neurological disorders (7%). Less common (< 5%) were cancer, heart disease, liver and kidney disease, digestive problems, migraines, autoimmune disorders, skin disease, diabetes, and arthritis/rheumatism. To assess youth activity limitations, parents were asked if their child had any long-term health problems or medical conditions that prevented/limited activities at school, play or other age appropriate activities (yes/no).

Among the sample of 859 youth, 285 had one or more chronic physical health problems in the period of time between baseline and 18 month follow-up. Excluded were youth with only mental health problems, learning difficulties or autism spectrum disorder. A total of 574 youth had no chronic physical health problem. Of the 285 youth with health problems, 94 had an activity limitation associated with their problem; 191 did not. Thirty-eight youth were reported to have an activity limitation but no health problem and were removed from the analysis. The remainder consisted of 536 youth with no health problem or activity limitation. The final sub-sample consisted of 821 youth with complete information on their mentoring status at 18 months and who satisfied our criteria for inclusion in one of the three health status categories. Youth missing mentoring status at the 18 month follow-up (*n* = 138) (997-859) and those with an activity limitation but no physical health problem (*n* = 38) did not differ significantly from the final sub-sample (821) on baseline demographics, personal characteristics, and environmental factors.

Measurement.

#### Dependent variables

Dependent variables (youth outcomes) included youth self-reports of social anxiety, depressed mood, and peer self-esteem. Social anxiety was assessed using the Social Anxiety Scale for Children-Revised (SASC-R) [21] which has18 anxiety-related items and four fillers. Sub-dimensions include: fear of negative peer evaluations (SAD-FNE) (8 items), social avoidance and distress with new situations or unfamiliar peers (SAD-NEW) (6 items), and generalized social avoidance and distress (SAD-G) (4 items). Five response options range from “not at all” to “all the time”. Sub-scales have good internal consistency, stability, and divergent and discriminate validity [22]. In this study, internal consistency of sub-scales at baseline and 18 months follow-up was as follows: SAD-FNE (*α* = .90, .94); SAD-NEW (*α* = .76, .81); and SAD-G (*α* = .68, .72).

Depressed mood was assessed using 8 items from the Center for Epidemiologic Studies Depression Scale (CES-DC) [23]. Four response options range from “not at all” to “a lot or all the time”. There is good internal consistency and retest stability and moderate support for concurrent validity [22]. For this study, internal consistency at baseline and 18 month follow-up was *α* = .76 and *α* = .83 respectively.

Peer self-esteem was measured using a 6 item abbreviated version of the peer sub-scale of the HARE Self-Esteem Scale [24]. Five response options range from “strongly agree” to “strongly disagree” (*α*s = .63 and .73 at baseline and follow-up, respectively).

#### Independent variable

Youth mentoring status was defined as the presence or absence of a BBBS mentoring relationship (i.e., mentored versus not mentored) between the baseline assessment and18 month follow-up. Non-mentored youth were the comparison group.

#### Moderator

Youth health status served as the hypothesized moderator in the SEM models (see Analytic Method section).

#### Covariates

Study covariates were chosen based on previous theory and research on youth mentoring [25]. Youth characteristics were: gender (1 = boys 0 = girls), age (continuous), living arrangements (two dummy-coded categories: living with a single biological parent only and living in other arrangements vs. a reference group of both biological parents), ethnic/racial minority status reported by the parent/guardian (1 = Aboriginal/First Nations/Metis/Inuit, African, Asian, and Hispanic Canadian 0 = all others), number of siblings at home (continuous), number of family moves (past five years) (continuous), and sought help from a mental health or social service professional in the past 12 months (1 = yes 0 = no). Parent/guardian characteristics included: age (continuous) and education (1 = < high school 0 = other) and family economic deprivation (count of: parent-reported gross annual household income < 20 K, government social assistance receipt, living in government subsidized dwelling). Other parent-reported covariates included: family functioning (*α* = .86), using 13-item general functioning sub-scale of McMaster Family Assessment Device, [26] parent depression (*α* = .92), using 20-item CES-D, [27] parent social anxiety (*α* = .92), using 17-item Social Phobia Inventory (*α* = .92), [28] and neighbourhood problems (*α* = .86), using 6 items from revised Simcha-Fagan Neighbourhood Questionnaire [29].

### Analytic method

Structural equation modeling (SEM) multiple groups analysis in M-Plus [30] was used to examine the relationship between mentoring status and outcomes (social anxiety, depressed mood, peer self-esteem) across health status groups. At the 18 month follow-up, each outcome was specified as a latent endogenous construct defined by three or more indicators and simultaneously regressed across the health status groups on: 1) youth mentoring status; 2) baseline scores for the same outcome; and 3) all covariates measured as observed variables. Baseline latent constructs and covariates were allowed to co-vary with youth mentoring status since youth eventually paired to a mentor may be differ from those without a mentor. Standard errors for estimated model parameters were adjusted for the nested data structure using the M-Plus Complex command. Missing data on outcomes at baseline and follow-up (2% and 26% respectively) were handled using Full Information Maximum Likelihood [31]. Goodness of fit was evaluated using the Chi-Square statistic and three standardized indices: Comparative Fit Index (CFI), Tucker-Lewis Index (TLI) and Root Mean Square Error of Approximation (RMSEA).

To reduce skewness on the latent construct indicators, construct items were parceled [32] into four sets for depressed mood (*α* = .78 and *α* = .85) and three for self-esteem (*α* = .61 and *α* = .72). The three SASC-R sub-scales formed the indicators of the social anxiety latent construct (*α* = .75).

To establish measurement invariance on the latent construct indicators, equality constraints were initially imposed on the factor loadings and intercepts across the three health status groups. Nested model comparisons **(**with and without constraints**)** based on chi-square difference values were conducted with non-significant values taken as evidence of measurement invariance. Minimal differences in model fit between constrained and unconstrained models revealed that the chosen indicators operated in a similar fashion across health status groups, and the decision was made to use the constrained models in subsequent analyses.

## Results

Table 1 shows youth and parent background characteristics entered as predictors and covariates in SEM models across health status groups. Youth health status was significantly correlated with youth living arrangements (χ^2^ (4, *N* = 821) = 11.60, *p* = .021) and use of mental health or social services in the previous 12 months (χ^2^ (2, *N* = 821) = 11.0, *p* = .004). Specifically, youth with one or more chronic health problems with activity limitation were significantly more likely than youth in the other health status groups to live with both biological parents and use services. Differences across health groups were also found for parent-reported family functioning (*F* (2, 818) = 3.10, *p* = .045), depressed mood (*F* (2, 818) = 7.34, *p* = .001), social anxiety (*F* (2, 818) = 6.12, *p* = .002), and neighbourhood problems (*F* (2, 818) = 5.66, *p* = .004). Post-hoc comparisons revealed significantly lower family functioning scores for youth with chronic health problems with activity limitation vs. those with chronic health problems without activity limitation, significantly higher scores on parent depressed mood and social anxiety for youth with chronic health problems with activity limitation versus both other groups, and higher scores on neighbourhood problems for youth with chronic health problems with activity limitation versus those without either chronic health problems or activity limitation.

**Table 1:** Description of Sample

Background Characteristics	Health 1^a^ (*n* = 191)	Health 2^b^ (*n* = 94)	Health 3^c^ (*n* = 536)
Youth			
Mentored (vs not)^d^	73.8	71.3	76.1
Gender (Boys) ^d^	54.5	42.6	50.4
Age^e^	9.55 (2.29)	10.05 (2.25)	9.74 (2.12)
Living Arrangements			
Both Biological Parents^d^	9.9	20.2	9.1*^a/b, b/c^
Single Biological Parent^d^	68.1	64.9	71.5
Other^d^	22.0	14.9	19.4
Ethnic Minority^d^	27.2	33.0	34.5
Mental Health/Social Service Use^d^	26.7	41.5	25.0*^a/b, b/c^
Number Siblings^e^	1.23 (1.39)	1.09 (1.04)	1.20 (1.26)
Number Family Moves^e^	1.53 (1.59)	1.78 (1.87)	1.67 (1.93)
Parents			
Age^e^	40.66 (8.87)	40.35 (8.45)	40.24 (8.82)
< High School Education^d^	14.1	13.8	17.2
Family Functioning^e^	52.12 (8.27)	49.62 (7.80)	51.37 (7.91)*^a/b^
Depressed Mood^e^	33.90 (10.46)	38.49 (11.29)	34.22 (10.25)*^a/b, b/c^
Social Anxiety^e^	15.37 (12.26)	19.45 (12.22)	14.87 (11.42)*^a/b, b/c^
Neighbourhood Problems^e^	8.29 (2.77)	9.04 (2.94)	8.04 (2.62)*^b/c^
Number Indicators of Economic Deprivation^e^	0.74 (.94)	0.88 (1.03)	0.77 (.98)

Standard deviations shown in brackets for characteristics measured on a quasi-continuous or continuous scale

*p < .05 for post-hoc comparisons

^a^one or more chronic physical health conditions and no activity limitation

^b^one or more chronic physical health conditions and an activity limitation

^c^neither a chronic physical health condition or activity limitation

^d^Estimated percentage

^e^Estimated mean value

**Fig. 1:**
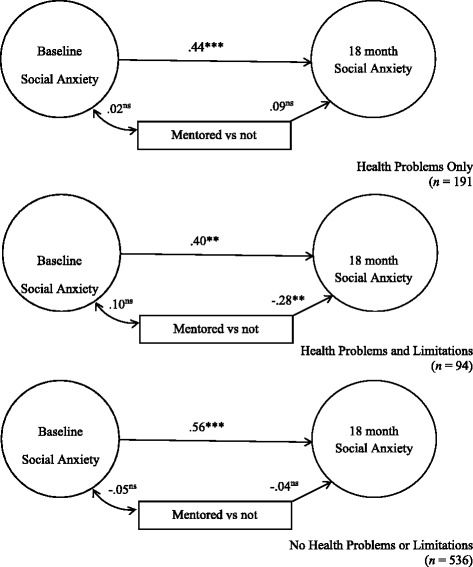
Social Anxiety. χ^2^ = 261.62, *df* = 211, *p* = .0101. RMSEA = .030, CI 90% .015-.041, *p* = .999, CFI = .972, TLI = .958. *p < .05, **p < .01, ***p < .001. Standardized structural coefficients adjusted for youth and parent baseline demographics and personal/environmental characteristics. Standard errors adjusted for design effects

In Fig. 1, a mentoring relationship was not significantly associated with improvements in social anxiety for youth with chronic health problems and no activity limitation and youth with no chronic health problem or activity limitation. Mentored youth were significantly more likely than non-mentored youth to report improvements in social anxiety for youth chronic health problems and activity limitation (γ = −.28, *p* < .01). The cross group comparison for this pathway between the chronic health problems and activity limitation group and chronic health problems and no activity limitation group (γ = .09, *ns*) revealed a statistically significant difference (χ^2^ (1) = 6.68, *p* = <.01).

**Fig. 2:**
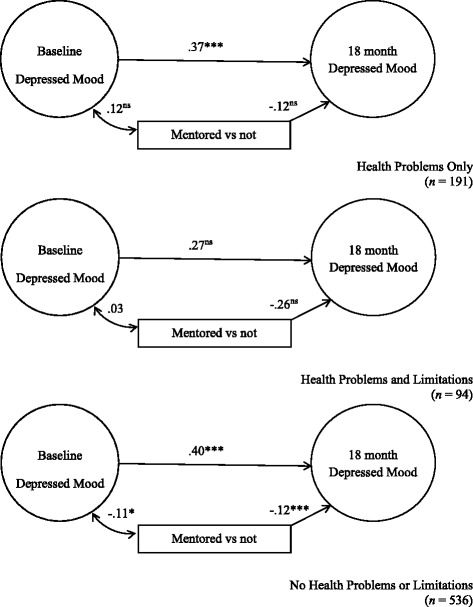
Depressed Mood. χ^2^ = 427.64, *df* = 339, *p* = .0008. RMSEA = .031, CI 90% .021-.040, *p* = 1.00, CFI = .954, TLI = .940. *p < .05, **p < .01, ***p < .001. Standardized structural coefficients adjusted for youth and parent baseline demographics and personal/environmental characteristics. Standard errors adjusted for design effects

In Fig. 2, being mentored was inversely correlated with symptoms of depressed mood for all health status groups but only reached statistical significance for youth with no chronic health problems or activity limitations (γ = −.12, *p* < .001).

**Fig. 3:**
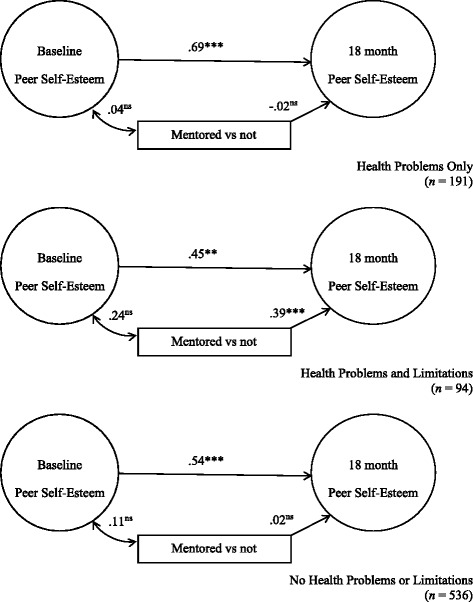
Peer Self**-**esteem. χ^2^ = 275.26, *df* = 211, *p* = .0019. RMSEA = .033, CI 90% .021-.044, *p* = .997, CFI = .926, TLI = .89. *p < .05, **p < .01, ***p < .001. Standardized structural coefficients adjusted for youth and parent baseline demographics and personal/environmental characteristics. Standard errors adjusted for design effects

In Fig. 3, mentored youth with chronic health problems and activity limitation reported stronger feelings of peer self-esteem than non-mentored counterparts (γ = .39, *p* < .001). The corresponding associations were statistically non-significant among the other groups. The difference between this estimated parameter and the parameters for the other health groups was statistically significant, (χ^2^ (1) = 4.14, *p* = <.05) and (χ^2^ (1) = 8.53, *p* = < .01).

## Discussion

Youth with one or more chronic physical health problems represented one third of study youth (23.3% chronic health problems and no activity limitation, 11.4% chronic health problems and activity limitation). Similar estimates have been obtained among Canadian youth [10].

In our sample, youth with one or more chronic physical health problems with or without activity limitations were similar to youth without chronic health problems or activity limitation on many background characteristics. Where differences were found, youth with chronic physical health problems and activity limitation differed most from other youth. These families may have and use more resources (e.g. living with both biological parents, use of mental health and social services) but may be limited as parents cope with their own difficulties with social anxiety, depressed mood and family functioning.

No significant differences in mentoring status were found across health status groups. This suggests getting matched within a mainstream mentoring program like BBBS is not different for youth with chronic health problems, with or without activity limitations, compared with youth without these difficulties.

Fewer symptoms of depressed mood was associated with having an adult mentor for all health groups but reached statistical significance only for youth with no chronic health problems or activity limitation. Others have also demonstrated an association between mentoring and improved mood [18, 33]. Mentored youth with chronic physical health problems and activity limitation were better off compared with non-mentored youth, exhibiting significantly fewer symptoms of social anxiety and higher levels of peer self-esteem. This association between mentoring and improved social anxiety and peer self-esteem was not found in the other health groups.

The association between having a mentor and reduced social anxiety and higher peer self-esteem among youth with chronic health problems and activity limitation may be related to the fact that their parents, who are coping with their own anxiety and mood difficulties, are unable to optimally support their children in these areas, so mentors play a key role. It is also possible that mentors become more empathic and sensitive when they are exposed to youth with chronic health problems that are perhaps more visible and that limit activities. It may be that training and match support by BBBS agencies was more specific or intensive for mentors matched with these specific youth. Others have suggested specific recruitment and training of mentors for special youth populations [34]. It may also be that mentoring works better for these youth with physical health problems and apparent limitations because these youth are more likely to be exposed to peer pressure, rejection and intimidation. The experience of connecting well with adults (e.g., when in hospital) and decreased experience with peers (e.g., related to poor school attendance) may also influence this finding. This may explain why the positive results associated with being mentored were specific to social interaction measures, not depressed mood.

Some limitations are noted. Although we demonstrated a significant relationship between being mentored and positive outcomes for youth with chronic health problems and activity limitation (social anxiety, peer self-esteem) and youth with no chronic health problem or activity limitation (mood), we cannot infer causality. While this study had a good response rate and national coverage, our sample was primarily from BBBS agencies in metropolitan centers so results may not generalize to youth residing in small towns or rural areas. Participant maturation from baseline to 18 months may also influence outcomes and is a threat to the validity of findings reflecting mentoring effects. Our measure of youth chronic health problems was dependent on parent reports without other confirmation (e.g., physician report) and excluded potential health moderators (e.g., type of health condition, persistence, associated pain). Future studies are needed to explore these additional factors in the context of delivering mentoring services to youth.

## Conclusions

Youth with chronic health problems and activity limitation may benefit from a BBBS one-to-one community mentoring relationship, particularly for social anxiety and peer self-esteem. All youth may experience a benefit to depressed mood associated with mentoring. For youth with chronic health problems, opportunities to be involved in main stream programs may reduce the focus on medical illness, decrease stigma and provide opportunities for skill building and social relationships. Practitioners working with youth with chronic health problems should consider recommending main stream mentoring programs as participation may improve how these youth fare.

## Abbreviations

BBBS: Big Brothers Big Sisters
CES-DC: Center for Epidemiologic Studies-Depression Scale
CFI: Comparative Fit Index
RMSEA: Root Mean Square Error of Approximation
SAD-FNE: Social Avoidance Distress-Fear of Negative Peer Evaluations
SAD-G: Social Avoidance Distress-Generalized
SAD-NEW: Social Avoidance Distress-New
SASC-R: Social Anxiety Scale for Children-Revised
SEM: Structural Equation Modelling
TLI: Tucker-Lewis Index
